# The Differences Between Dopamine Agonist-Resistant and -Non-Resistant Prolactinomas: Are There Any Predictors of a Good Response?

**DOI:** 10.3390/biomedicines14010234

**Published:** 2026-01-21

**Authors:** Maria Komisarz-Calik, Anna Bogusławska, Aleksandra Gamrat-Żmuda, Mari Minasyan, Beata Piwońska-Solska, Jacek Kunicki, Grzegorz Zieliński, Agata Faron-Górecka, Alicja Hubalewska-Dydejczyk, Aleksandra Gilis-Januszewska

**Affiliations:** 1 Department of Endocrinology, Jagiellonian University Medical College, 30-688 Krakow, Poland; maria.komisarz@doctoral.uj.edu.pl (M.K.-C.); anna1.boguslawska@uj.edu.pl (A.B.); aleksandra.gamrat@gmail.com (A.G.-Ż.); mari.minasyjan@gmail.com (M.M.); beata.piwonska-solska@uj.edu.pl (B.P.-S.); alahub@cm-uj.krakow.pl (A.H.-D.); 2 Doctoral School of Medical and Health Sciences, Jagiellonian University Medical College, 31-530 Krakow, Poland; 3 Maria Sklodowska-Curie National Research Institute of Oncology, 02-781 Warsaw, Poland; jacek.kunicki@nio.gov.pl; 4 Department of Neurosurgery, Military Institute of Medicine, 04-141 Warsaw, Poland; gzielinski@wim.mil.pl; 5 Maj Institute of Pharmacology, Polish Academy of Sciences, 31-343 Krakow, Poland; gorecka@if-pan.krakow.pl

**Keywords:** prolactinoma, dopamine agonist (DA) resistance, predictive factors, cabergoline, tumor shrinkage

## Abstract

**Background/Objectives**: Dopamine agonists (DAs) are the first-line therapy for prolactinomas; however, a subset of patients exhibits resistance or incomplete response. **Methods**: This retrospective study included 85 of 125 eligible consecutive patients with prolactinoma who were treated with DA, followed for a median of 52.0 (31.5–86.8) months. Clinical, biochemical, and radiological parameters were analyzed at baseline and at 6 and 12 months. Resistance was defined as failure to normalize serum prolactin concentration (PRL) or achieve ≥ 30% reduction in tumor maximal diameter after standard DA therapy. Logistic regression analyses were performed to identify predictors of DA resistance and treatment response. **Results**: The cohort comprised 54 males (63.5%) and 31 females (36.1%), with a mean age of 41.5 ± 17.2 years. In total, 22.4% had giant prolactinomas. After 6 months of treatment, 24.7% achieved PRL normalization, and 29.4% demonstrated ≥ 50% reduction in tumor volume. At 12 months, PRL normalized in 40% of patients, and a ≥50% volume reduction was observed in 41.2%. DA-resistant patients, compared to DA-non-resistant, were predominantly men (80.0% vs. 56.7%, *p* = 0.042), with a higher proportion of giant adenomas (44.0% vs. 13.3%, *p* = 0.002) and significantly higher baseline PRL (2000.000 ng/mL vs. 478.985 ng/mL, *p* = 0.012). Early reduction in maximal tumor diameter at 6 months predicted a favorable therapeutic response at 12 months (aOR = 1.156; 95% CI = 1.001–1.335, *p* = 0.049). **Conclusions**: Male sex, higher baseline PRL, and larger tumor size can be predictors of DA resistance. On the other hand, early radiological tumor shrinkage may predict favorable treatment outcomes. However, new markers of DA resistance, particularly molecular ones, should be identified.

## 1. Introduction

Prolactinomas are the most common type of pituitary tumors, accounting for approximately 50% of pituitary adenomas [1]. In the great majority of cases, they respond well to dopamine agonist (DA) therapy; however, about 10–20% of macroprolactinomas and 5–10% of microprolactinomas are resistant to DA therapy [2,3]. Among DAs, cabergoline is the most effective and most frequently used; the overall prevalence of DA resistance is approximately 10% with cabergoline and 20–30% with bromocriptine [2]. According to the 2011 guidelines, resistance was defined as failure to achieve normoprolactinemia and an inability to reduce tumor size by 50% despite the maximum tolerated dose of DA [4]. However, the approach to the concept of resistance has evolved over the years, and according to the newest 2023 Pituitary Society International Consensus Statement, resistance is defined as a lack of normalization of prolactin serum concentration (PRL) or lack of relevant mass shrinkage (≥30% reduction in maximum diameter) when treated with standard dopamine agonist doses (7.5–10 mg per day of bromocriptine or 2.0 mg per week of cabergoline) for at least 6 months [3]. Variability in response to DA treatment has been attributed to several factors, including the initial tumor size. Cabergoline achieves prolactin normalization in approximately 80–90% of patients with prolactinomas; however, remission rates decline with increasing adenoma size, reaching 70–80% in macroprolactinomas [5,6]. In giant prolactinomas (macroprolactinomas measuring > 4 cm), DA efficacy is high, with normalization of serum prolactin levels in 60% and reduction in tumor volume in 74% of individuals [3].

The mechanism of resistance to DA therapy remains unclear; however, certain factors, such as male sex [7], young age, tumor invasiveness, cavernous sinus invasion, and higher Ki-67 expression, are also associated with tumor aggressiveness. From a molecular perspective, lower dopamine receptor 2 (D2) expression [7] and lower estrogen receptor (ER) expression, especially ERα66 and ERα36 [8], are associated with DA resistance. Furthermore, some variants of the prolactin receptor (PRLR) [9] and tumor hypointensity on magnetic resonance imaging (MRI) [10] have been associated with DA resistance.

The treatment of a DA-resistant prolactinoma can be challenging, and a multimodal approach is often required. In case of a bromocriptine-resistant patient, switching to cabergoline treatment is advised [3,11]. In patients treated with a standard dose of cabergoline, dose escalation is recommended up to the maximum tolerated dose [3]. In cases of refractory prolactinomas necessitating tumor control, pituitary surgery constitutes the following line of therapy [3]. Another treatment option is radiotherapy, in particular for patients with poor mass shrinkage after DA therapy with non-resectable residual tumor tissue [3]. The great majority of refractory prolactinomas will respond to multimodal treatment; however, some patients, particularly those with aggressive prolactinomas, may require alternative therapy, such as pasireotide [12,13,14], temozolomide [3,15], or other experimental therapies [12].

Although several clinical and molecular markers associated with DA resistance have been proposed, data are inconsistent and often derived from small or highly selected cohorts. Few studies have evaluated early radiological or biochemical predictors that may help anticipate treatment response. Robust, real-world data from contemporary cohorts are still limited, particularly regarding the predictive value of early tumor shrinkage under standard cabergoline therapy. Identifying the factors underlying DA resistance is crucial when planning therapy for patients with prolactinoma, as it enables the prediction of treatment response at the time of treatment planning and initiation.

Therefore, we aimed to investigate the frequency of DA resistance at 6 and 12 months of therapy and to identify clinical and radiological predictors associated with resistance, using the current consensus definition (lack of prolactin normalization or lack of ≥30% reduction in maximal tumor diameter after ≥6 months of standard-dose DA therapy).

## 2. Materials and Methods

**Figure 1:**
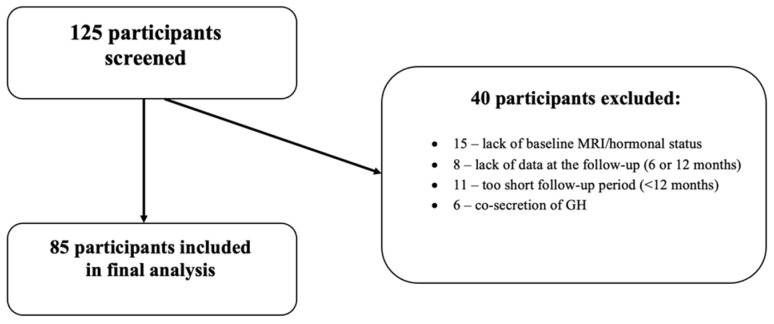
Flowchart of the patients included in a study.

We retrospectively analyzed the medical records of 125 consecutive patients with prolactinomas who were diagnosed and treated at a tertiary endocrinological referral center in southern Poland between 2014 and 2024. The inclusion criteria were patients aged 18 years or older with a confirmed diagnosis of prolactinoma and a minimum follow-up period of one year under cabergoline treatment. We excluded patients with clinical or biochemical suspicion of growth hormone (GH) co-secretion, patients with hyperprolactinemia without pituitary adenoma, patients after pituitary surgery with negative immunohistochemical staining for PRL, and patients with incomplete data at baseline, at 6-month follow-up, or at 12-month follow-up. Consequently, 85 patients who met these criteria were included in the study (Figure 1).

Demographic data (age and sex), symptoms, hormonal levels, tumor characteristics (including invasion of the sphenoid and cavernous sinuses), and the KNOSP classification were collected, along with follow-up data. At 6 months, we analyzed follow-up data and classified patients as responders, partial responders, or resistant to DA therapy according to current guidelines [3]. **Responders** were defined as patients who achieved normopolactinemia and at least a 30% reduction in the maximal tumor diameter after at least 6 months of DA treatment, irrespective of the administered DA dose. Patients were classified as **resistant to DA** therapy when they did not show prolactin normalization or at least a 30% reduction in the maximal tumor diameter after at least 6 months of DA therapy with standard (7.5–10 mg per day of bromocriptine or 2.0 mg per week of cabergoline) or maximally tolerated DA doses. Patients who did not meet the criteria for DA resistance and could not be classified as responders were classified as **partial responders**: (1) patients achieving normoprolactinemia without at least 30% reduction in the maximal tumor diameter treated with lower-than-standard DA doses (<7.5 mg per day of bromocriptine or <2.0 mg per week of cabergoline), (2) patients not achieving normoprolactinemia with at least 30% reduction in the maximal tumor diameter treated with lower-than-standard DA doses, (3) patients not achieving normoprolactinemia and not achieving at least 30% reduction in the maximal diameter, treated with lower-than-standard DA doses.

Prolactin concentration (PRL) was expressed in ng/mL. Changes in prolactin levels at follow-up were defined as the delta (Δ) between baseline and follow-up PRL concentrations, calculated using the following formula: ΔPRL = (PRL follow-up − PRL baseline). The maximum cabergoline dose was defined as the highest dose used during the 6- or 12-month follow-up period and was expressed as the median (Me) and interquartile range (IQR).

Tumor volume (TV) was calculated using the ellipsoid formula: A × B × C × Π/6 (A—width; B—height; C—depth of the tumor). Changes in tumor volume were defined as percentages according to the formula: (TV baseline − TV follow-up)/TV baseline × 100. Moreover, we analyze changes in the maximal tumor diameter (MTD) and calculate the percentage reduction in the maximal tumor diameter (MTD baseline − MTD follow-up)/MTD baseline × 100. MRI scans were evaluated by an experienced neuroradiologist using a standardized pituitary protocol. Measurements were performed consistently across time points to reduce inter-scan variability.

We performed a comparative analysis between patients with resistant prolactinomas (as determined at 6-month follow-up) and those without resistance (responders and partial responders) to identify potential factors associated with resistance.

Statistical analysis was performed using IBM SPSS Statistics software (version 29). Normally distributed data were presented as mean ± standard deviation (SD), while non-normally distributed data were presented as median (Me) and (quartile 1 − quartile 3 (q1 − q3)). Considering the non-normal distribution of the continuous variables, the Mann–Whitney U test was used. Categorical variables were expressed by the frequencies and percentages (%) and compared using the χ^2^ test or with Fisher’s exact test. Univariate and multivariate logistic regression analyses were conducted to identify potential predictors of resistance to DA therapy at the first-year follow-up. Results from the multivariate logistic regression analyses were presented as odds ratios (OR) with 95% confidence intervals (CI). A *p*-value of <0.05 was considered statistically significant. The study was approved by the Ethics Committee of Jagiellonian University (approval number: 118.0043.1.231.2024). Patient consent was not required because the study was retrospective.

## 3. Results

### 3.1. Baseline Characteristics

A total of 85 patients with prolactinoma were included in the final analysis. Most patients (63.5%) were male. The mean age at diagnosis was 41.52 ± 17.19 years. The dimensions of the tumor showed significant variability (the median maximum diameter was 22.0 mm (min = 2.0 mm, max = 98.0 mm); additionally, 19 tumors (22.4%) were classified as giant prolactinomas. The median tumor volume was 3364.65 (IQR = 630.28–14,168.62) mm^3^. The baseline median prolactin concentration was 545.7 (min = 11.4; max = 51,000.0) ng/mL.

The most common symptoms were headache (69.4%), visual field disturbances (35.3%), and libido loss (18.8%). Furthermore, 67.7% of women reported irregularity of menstrual cycles and 16.5% galactorrhea. Among men, 27.8% complained of erectile dysfunction and 9.3% noticed gynecomastia. Central hypogonadism occurred in 76.5% of patients. Insufficiencies of other pituitary axes were less common; both corticotropic- and thyroid-axis insufficiency were observed in approximately one-quarter of patients. In our cohort, no cases of panhypopituitarism were observed (Table 1).

In 69 patients (81.2%), invasion of the cavernous sinuses was described on MRI; in 61.4%, invasion of the sphenoid sinuses was noted, and in 44.6%, the optic chiasm was compressed. According to KNOSP grade, the most prominent were grades 2 (23.5%) and 3 (25.49%). At baseline, 22.4% of the tumors comprised cystic components (Table 1).

#### 3.1.1. Evaluation at 6 Months

At 6 months of DA treatment, only 24.7% of patients normalized prolactin levels. In 29.4% of patients, we observed a ≥50% reduction in tumor volume, and in 18.8% of individuals, we noticed ≥ 30% reduction in the maximal tumor diameter. The median prolactin concentration after 6 months was 47.4 (IQR = 14.45 − 241.35) ng/mL. The median delta prolactin concentration after 6 months was 391.42 (IQR = 45.05 − 1476.85) ng/mL. The median reduction in tumor volume was 23.15 (IQR = 0.0 − 57.11) %, and the median reduction in maximal tumor diameter was 7.69 (IQR = 0.0 − 26.36) %. The maximal doses of cabergoline used during the 6-month follow-up ranged between 0.25 mg/week and 4.0 mg/week, with a median of 1.0 mg/week (IQR = 0.75 − 2.0). In total, 29.4% of individuals were categorized as resistant to DA, 7.1% as responders, and 63.5% as partial responders (Table 2).

#### 3.1.2. Evaluation at 12 Months

Analyzing the response for the treatment after 12 months of DA treatment, we observed that only 40.0% of patients normalized the prolactin concentration, and in 41.2% of patients we observed a ≥50% reduction in tumor volume; however, in 67.1% of patients we observed some volume reduction, and in 15.7%, tumor growth. In 28.2% of patients, we observed a ≥ 30% reduction in maximal tumor diameter. The median prolactin concentration after 12 months was 24.8 (IQR = 6.85 − 61.15) ng/mL, and the median delta prolactin concentration after 12 months was 457.97 (IQR = 105.4 − 1995.95) ng/mL. The median reduction in tumor volume was 43.7 (IQR = 17.27 − 78.25) %, and the median decrease in maximal tumor diameter was 15.4 (IQR = 0.00 − 33.33) %. The maximal doses of cabergoline used during the 12-month follow-up ranged between 0.25 mg/week and 4.5 mg/week, with a median of 1.0 mg/week (IQR = 0.75 − 2.0). After 12 months of treatment, 10.6% of patients were classified as responders, 57.6% as partial responders, and 31.8% as DA-resistant (Table 2). We observed that three patients classified as partially responsive at the 6-month follow-up became responsive at the 12-month follow-up, while two patients classified as partially responsive at the 6-month follow-up became resistant.

#### 3.1.3. Long-Term Follow-Up

The duration of follow-up was highly heterogeneous, with a median of 52.0 (IQR = 31.5 − 86.75) months (Table 1). Despite a relatively long follow-up period, prolactin normalization was achieved in 55.3% of patients, and ≥50% tumor volume reduction in 51.8%. In 69.4% of patients, we observed some reduction in tumor volume, and in 36.5%, we observed ≥ 30% reduction in maximal tumor diameter. We observed an increase in response rate to 25.9%. Five patients who were DA-resistant at 12 months became responsive at the last follow-up; two patients who were DA-resistant at 12 months became partially responsive; and the DA resistance rate in prolactinomas decreased to 23.5%. Moreover, eight patients classified as partially responsive at 12 months became responsive at the last follow-up, reducing the percentage of partial responders to 50.6% (Table 2).

### 3.2. Other Methods of Treatment

Surgical treatment was performed on 15.5% (13/85) of patients (Table 1). One patient experienced apoplectic intratumoral hemorrhage. In one patient, the surgery was performed twice; however, due to the inoperable character of the tumor, the patient still needs therapy, and he is treated with cabergoline and Pasireotide LAR to control the prolactin levels and tumor growth. The other patient, with an aggressive giant prolactinoma and a total follow-up of 12 years, underwent three non-radical neurosurgical procedures and was treated with Lanreotide LAR, Temozolomide, and then palliative radiotherapy; however, the patient died within a few days of radiotherapy.

#### 3.2.1. Comparison of Resistant and Non-Resistant Prolactinomas

We conducted a comparative analysis of patients with resistant and non-resistant prolactinomas to identify potential factors associated with DA resistance. Patients with resistant prolactinomas had significantly higher baseline prolactin concentrations (2000.000 ng/mL vs. 478.985 ng/mL, *p* = 0.012), larger tumors (median maximal diameter: 30.0 mm vs. 19.0 mm, *p* = 0.012), with higher basal volume (10,306.540 mm^3^ vs. 2111.157 mm^3^, *p* = 0.014) and were more frequently male (80.0% vs. 56.7%, *p* = 0.042). The percentage of giant tumors among resistant prolactinomas was higher than among non-resistant prolactinomas (44.0% vs. 13.3%, *p* = 0.002), and suprasellar invasion at baseline MRI was more common (52.0% vs. 28.3%, *p* = 0.037). Additionally, at 6 and 12 months and at the last follow-up, the prolactin concentration was substantially lower in the non-resistant prolactinomas (29.805 ng/mL vs. 148.000 ng/mL, *p* < 0.001), (14.750 ng/mL vs. 60.000 ng/mL, *p* < 0.001), and (13.150 ng/mL vs. 32.100 ng/mL, *p* = 0.005), respectively. At the last visit, the median delta prolactin was significantly higher in resistant than in non-resistant prolactinomas (1950.000 ng/mL vs. 424.535 ng/mL, *p* = 0.019). Patients with resistant prolactinomas were administered higher doses of cabergoline during the 6-month follow-up (median: 2.0 mg/week vs. 1.0 mg/week; *p* < 0.001) as well as during the 12-month follow-up (median: 2.5 mg/week vs. 1.0 mg/week; *p* < 0.001) (Table 3). The maximal doses of cabergoline used during the 6-month follow-up ranged from 2.0 mg/week to 4.0 mg/week in the resistant group, whereas in the non-resistant group, they ranged from 0.25 mg/week to 4.0 mg/week. During the 12-month follow-up, the maximal dose of cabergoline ranged between 2.0 mg/week and 4.5 mg/week in the resistant group and between 0.25 mg/week and 4.0 mg/week in the non-resistant group.

#### 3.2.2. Sex Differences

Given the widely recognized difference in clinical course between women and men with prolactinomas, we conducted a sex-stratified analysis. The median baseline prolactin concentration was higher in men (976.68 ng/mL vs. 204.35 ng/mL, *p* < 0.001). At 6 and 12 months and at the last follow-up, we did not observe significant differences in prolactin concentration. Tumors in men had higher baseline and 12-month-follow-up volumes (7961.86 mm^3^ vs. 605.67 mm^3^, *p* < 0.001), (2333.16 mm^3^ vs. 367.6 mm^3^, *p* < 0.001). In men, the cabergoline dose was considerably higher at 6 and 12 months (1.0 mg/week (IQR = 1.0 − 2.0 mg/week) vs. 1.0 mg/week (IQR = 0.5 − 1.5 mg/week), *p* = 0.013), (1.5 mg/week (IQR = 1.0 − 2.5 mg/week) vs. 1.0 mg/week (IQR = 1.0 − 1.5 mg/week), *p* = 0.002), respectively. The resistance to DA after 6 and 12 months of therapy was significantly more frequent in men than in women (37.0% vs. 16.1%, *p* = 0.042; 40.7% vs. 16.1%, *p* = 0.019, respectively). In a subsequent follow-up, these differences were not statistically significant (Table 4). However, the analysis of the groups of resistant and non-resistant prolactinomas showed no difference in cabergoline dose between males and females.

#### 3.2.3. Correlations

At baseline, higher prolactin concentration positively correlated with higher tumor maximum diameter (rho = 0.449; *p* < 0.001) and tumor volume (rho = 0.395; *p* < 0.001). Higher cabergoline doses correlated positively with higher maximal dimension of the tumor (rho = 0.366; *p* < 0.001) and tumor volume (rho = 0.320; *p* = 0.003).

#### 3.2.4. Association with Resistance and Response to DA

Firstly, we performed univariate logistic regression to identify predictors of resistance and response at 6 and 12 months.

To find predictors of resistance at 6 months of age, sex, baseline prolactin concentration, baseline tumor volume, and baseline maximal tumor diameter were evaluated.

To identify predictors of resistance at 12 months, in addition to age, sex, baseline prolactin concentration, baseline tumor volume, and baseline maximum tumor diameter, the % tumor volume reduction at 6 months, % maximum tumor diameter reduction at 6 months, and delta prolactin concentration between baseline and 6 months were evaluated. At 6 months, male sex (OR = 3.059; 95% CI = 1.013–9.236, *p* = 0.047) and baseline maximum tumor diameter (OR = 1.044; 95% CI = 1.016–1.073, *p* = 0.002) were predictive of resistance to DA. Similarly, at 12 months, male sex (OR = 3.575; 95% CI = 1.190–10.743, *p* = 0.023) and baseline maximal tumor diameter (OR = 1.047; 95% CI = 1.018–1.076, *p* = 0.001) were predictive of DA resistance (Table 5). At 6 months, the baseline tumor volume (OR = 1.000; 95% CI = 1.000–1.000, *p* = 0.005) and at 12 months, baseline tumor volume (OR = 1.000; 95% CI = 1.000–1.000, *p* = 0.004) and baseline prolactin concentration (OR = 1.000; 95% CI = 1.000–1.000, *p* = 0.008) were significantly associated with resistance; however, considering the fact that the estimated odds ratio per unit increase was numerically close to unity, small, single-unit changes in tumor volume [mm^3^] and prolactin concentration [ng/mL] are less likely to be clinically meaningful.

We evaluated the same variables to identify predictors of response at 6 and 12 months. Only the reduction in the maximum tumor diameter at 6 months was predictive of a good response at 12 months (OR = 1.075; 95% CI: 1.032–1.121, *p* < 0.001). In the next step, the multivariable logistic regression analysis was performed, and the best model to predict the response to DA at 12 months showed that a reduction in maximum diameter at 6 months (aOR = 1.156; 95% CI = 1.001–1.335, *p* = 0.049) was a good predictor of the response. Other variables included in the model, such as age at onset (*p* = 0.927), sex (*p* = 0.785), reduction in tumor volume at 6 months (*p* = 0.720), and delta of PRL concentration at 6 months (*p* = 0.070), did not reach statistical significance.

## 4. Discussion

In this study, we examined treatment outcomes and follow-up of 85 patients with prolactinoma treated at a tertiary endocrinological referral center in southern Poland, with a focus on response to DA therapy. Over 60% of patients included in the study were male, even though prolactinomas are mainly seen in women [1]. A possible explanation is that tertiary referral centers usually admit patients with more advanced disease stages, larger tumors—including giant prolactinomas—and those resistant to standard treatments.

Evaluating the response to DA therapy, we observed that after 6 months, serum prolactin levels normalized in 24.7% of patients, and the median tumor volume reduction was 23.15%. After 12 months, these rates rose to 40.0% and 43.7%, respectively. These results are consistent with previous reports, indicating that 10–20% of macroprolactinomas and 5–10% of microprolactinomas show resistance to DA [13,14].

At long-term follow-up (median 52 months), normalization of prolactin was achieved in 55.3% of patients and tumor shrinkage in 69.4%, demonstrating that the efficacy of DA therapy improves over time, and that delayed responses are common, particularly in large or invasive prolactinomas. However, according to the guidelines, a significant subgroup remained resistant or only partially responsive. At extended follow-up, 23.5% of patients were classified as resistant, 25.9% achieved a complete response to DA, and 50.6% achieved only a partial response [3]. After 12 months of treatment, 10.6% of patients were classified as responders, 57.6% as partial responders, and 31.8% as DA-resistant. Notably, the proportion of DA-resistant cases decreased to 23.5% at long-term follow-up, reflecting the presence of late responders who achieved biochemical or radiological improvement beyond the first year of therapy. Although the percentage of patients with DA resistance in our study was comparable to that reported in the literature, a substantial proportion of patients were classified as partial responders.

A possible explanation is the relatively low cabergoline doses, with a median of 1.0 mg/week in the study, particularly compared to doses used in resistant prolactinomas (up to 3.5–7 mg/week in some studies). During subsequent follow-up visits, we identified 58 patients (54 partial responders and 4 responders) at 6 months and 56 patients (49 partial responders and 7 responders) at 12 months who were treated with a cabergoline dose of < 2.0 mg/week. Among patients with DA resistance, we identified only one patient who was intolerant of doses of cabergoline exceeding 2.0 mg/week. A higher proportion of individuals with intolerance was observed in the partially responsive group, in which above 90% were receiving doses below 2.0 mg/week, which likely reflects intolerance rather than insufficient treatment intensity. Clearly, we observed that patients resistant to DA had significantly higher doses of DAs during the 6- and 12-month periods of follow-up (6 months: min: 2.0 mg/week to max: 4.0 mg/week in the resistant group vs. min: 0.25 mg/week to max: 4.0 mg/week in the non-resistant group; 12 months: min: 2.0 mg/week and max; 4.5 mg/week in the resistant group vs. min: 0.25 mg/week and max: 4.0 mg/week in the non-resistant group). However, even in the resistant group, cabergoline doses were relatively low, which may have affected the proportion of resistant and partially responsive prolactinomas.

Another critical factor that could influence the response to DA is the presence of a cystic component in the tumor. In our study, we observed a relatively high prevalence (22.4%) of cystic components in tumors at baseline, which may reflect a high resistance rate. The presence of cystic components in the tumour should be distinguished from the predominantly cystic prolactinomas, which are filled with fluid in more than 50% of their volume [13], and are less susceptible to DA therapy [14]; however, in patients without urgent need of optic chiasm decompression, the DA should be considered and may induce the reduction of the cyst [3,15]. On the other hand, DA treatment may alter tumour histology, leading to cellular degeneration and fibrosis [16,17]; thus, initially solid prolactinomas can undergo cystic degeneration during DA therapy [18,19]. Interestingly, cystic prolactinomas have lower baseline prolactin levels [20]; therefore, one may hypothesize that tumor transformation into a more fluid-filled form is associated with a decrease in prolactin concentration and, consequently, a better response to DA.

Another factor possibly influencing resistance to DA is tumor intensity on T2-weighted MRI. Kreutz et al. found that lesions with hypointensities on T2-weighted MRI are almost exclusively encountered in men and correspond to high-density spots on computed tomography (CT). These patterns, in agreement with the literature, most likely reflects the presence of amyloid spheroid deposits. could translate to an amyloid spheroid deposit [21]. However, these data are limited, and the underlying histologic and structural characteristics that determine T2 intensity are poorly understood [22].

In further analysis of potential factors associated with resistance, we found that DA-resistant prolactinomas exhibited significantly higher baseline prolactin levels (2000.0 ng/mL vs. 478.985 ng/mL, *p* = 0.012), larger diameters (30.0 mm vs. 19.0 mm, *p* = 0.012), with greater baseline tumor volume (10306.540 mm^3^ vs. 2111.157 mm^3^, *p* = 0.014), male predominance (80.0% vs. 56.7%, *p* = 0.042), a higher proportion of giant adenomas (44.0% vs. 13.3%, *p* = 0.002) and suprasellar invasion at baseline MRI (52.0% vs. 28.3%, *p* = 0.037).

Men are more likely to present with macroprolactinomas or giant tumors and to show poorer therapeutic response [23,24]. In our study, giant tumors accounted for 22.4% of all cases, and resistance was more frequent in giant prolactinomas. In 82.1% of individuals, cavernous sinus invasion was noted, with a similar frequency between resistant (80.0%) and non-resistant tumors (81.7%); however, suprasellar invasion of the prolactinoma was substantially prevalent in resistant tumors (52.0% vs. 28.3%, *p* = 0.037). This relatively high frequency of cavernous sinus invasion and suprasellar infiltration most likely reflects that patients were recruited from a tertiary referral center, where individuals with more complex disease courses or more advanced stages of the condition are typically managed. These observations—male predominance and a higher incidence of suprasellar invasion are consistent with data from previous studies, including those by Delgrange et al., which showed that male sex and tumor invasiveness were associated with resistance [25].

Several studies have shown that DA is effective in managing giant prolactinomas. In a longitudinal analysis of 84 Swedish patients with giant prolactinomas, prolactin normalization was achieved in 55%, significant tumor reduction occurred in 69%, and a combined response (normalized prolactin and significant tumor reduction) was seen in 43% [26]. Espinosa et al. noted that the combined goal of prolactin normalization and tumor reduction of more than 50% was achieved in 55% of patients with giant prolactinomas and 66% of patients with macroprolactinomas [27].

A reduction in prolactin levels or tumor size within the first year of therapy can predict response to DA at follow-up [26,28,29]. Our findings extend this evidence by demonstrating that maximal tumor diameter reduction at 6 months is a particularly strong predictor of therapeutic success at 12 months. Lee et al. observed that a ≥25% tumor volume reduction at the third month is a good predictor of final volume reduction [28]. Similarly, Biagetti et al. found that ≥30% tumor shrinkage between the third and fourth months is a predictor of long-term response to DA treatment [29]. Another approach was presented by Kim et al. The researchers demonstrated that a prolactin level < 1 ng/mL at the third month increased the likelihood of subsequent tumor shrinkage of 20% or more [30]. Finally, Himonakos et al. noted that prolactin levels < 2x the upper limit of normal or a tumor diameter reduction of up to 20% during the first year of follow-up predicted the composite goal at final follow-up [26].

In the study, we conducted a logistic regression; in univariate analysis, male sex (OR = 3.059; 95% CI = 1.013–9.236, *p* = 0.047), (OR = 3.575; 95% CI = 1.190–10.743, *p* = 0.023) and baseline maximum tumor diameter (OR = 1.044; 95% CI = 1.016–1.073, *p* = 0.002), (OR = 1.047; 95% CI = 1.018–1.076, *p* = 0.001) were linked to DA resistance at 6 and 12 months. In addition, baseline tumor volume (OR = 1.000; 95% CI = 1.000–1.000, *p* = 0.004) and baseline prolactin concentration (OR 1.000; 95% CI = 1.000–1.000, *p* = 0.008) were significantly associated with resistance at 12 months. Because the estimated odds ratio per unit increase was numerically close to unity, single-unit changes in tumor volume [mm^3^] and prolactin concentration [ng/mL] are less likely to be clinically meaningful. However, our findings suggest that higher prolactin concentrations may be associated with DA resistance, as patients with resistant prolactinomas had substantially higher prolactin levels at all follow-up visits.

Additionally, in multivariable logistic regression analysis, the percentage of the reduction in maximum diameter at 6 months (aOR = 1.156; 95% CI = 1.001–1.335, *p* = 0.049) was a significant predictor of a good therapeutic response after 12 months of DA therapy. This finding supports earlier evidence that early radiographic improvement is associated with favorable long-term outcomes [29].

Our findings highlight the importance of early detection of patients at high risk of DA resistance—especially men with large, invasive tumors and very high baseline prolactin levels—as these individuals may benefit from earlier dose escalation or timely consideration of multimodal therapy. In cases with aggressive tumor behavior or in patients with resistance to DA, the multimodal treatment approach is necessary, including, besides the DA treatment, surgery (preferably transsphenoidal surgery) and radiotherapy [3]. In patients with aggressive tumors, surgery is recommended when, due to the sellar mass effect or apoplexy, rapidly progressive vision loss occurs, or in patients with intolerance or resistance to long-term DA therapy [3]. Moreover, alternative pharmacotherapies such as pasireotide, temozolomide, and immune checkpoint inhibitors are increasingly used in clinical practice [3,31,32,33,34,35]. Temozolomide (an oral alkylating chemotherapeutic agent) is recommended in aggressive prolactinomas with documented persistent tumor growth, despite exhausting all treatment modalities [3,31,35]. After temozolomide failure, the immune checkpoint inhibitors can be introduced [3,31]. Pasireotide (a second-generation somatostatin analog with high affinity for multiple somatostatin receptor subtypes (SST), particularly SST1, SST2, SST3, and SST5) is also effective at achieving biochemical and tumor control in DA-resistant prolactinomas by inducing cystic degeneration and tumor cell necrosis [32,33,34].

It is crucial to consider genetic and molecular biomarkers, such as somatic SF3B1^R625H^ mutations, which have been identified in 20% of prolactinomas and are associated with higher serum prolactin levels and potentially more aggressive behavior than prolactinomas without this mutation [3,36]. Although prolactinomas are very rarely associated with germline mutations, when present, these are typically associated with multiple endocrine neoplasia type 1 (*MEN1*) or a mutation in the *AIP* (aryl hydrocarbon receptor interacting protein) gene with disease onset at a young age and more aggressive behavior and resistance to therapy [3,37,38].

As presented in our study, the gradual improvement in biochemical and radiological outcomes over time underscores the dynamic nature of DA responsiveness. It supports caution against labeling patients as resistant too early, particularly in those with large or invasive tumors who may exhibit delayed—but clinically meaningful—responses. Additionally, the persistent sex-related differences in tumor biology and treatment response merit further mechanistic investigation.

### Limitations of the Study

The first issue is the study’s retrospective design, which limits the ability to control for confounding variables. Additionally, the relatively long interval between diagnosis and the last follow-up in some patients, along with variations in diagnostic and therapeutic approaches over time, may affect comparability. Missing data were also related to the study’s retrospective nature. Another factor is that patients were recruited from a tertiary referral center that admits patients with larger tumors and resistance to standard treatments. Furthermore, the lack of standardized definitions of DA resistance and dose thresholds in the literature hampers comparison across studies. This study also has the limitation of a relatively small number of patients and a quite heterogeneous cohort, including both microprolactinomas and macroprolactinomas. However, by including both smaller and larger tumors, the authors aimed to demonstrate that resistance to DA therapy can occur in both micro- and macroprolactinomas, and excluding either could limit the study’s findings. Future research should aim to identify molecular biomarkers, such as D2 receptor expression, ERα isoforms, and nerve growth factor receptor (NGFR) pathways, to predict treatment outcomes and guide personalized therapy.

## 5. Conclusions

Our data confirm that male sex, larger tumor size, and higher baseline prolactin levels are significantly associated with resistance to dopamine agonist therapy. Early radiological improvement (reduction in maximal tumor diameter at 6 months) appears to be a reliable marker of treatment success, corresponding to a good response to DA treatment. Moreover, our data suggest that the possible cause of resistance or partial response to DA therapy is that DA doses used in treatment are too low, often <2.0 mg of cabergoline per week. However, the response to DA is heterogeneous, and known clinical and radiological factors provide only partial predictive value for drug resistance. This reinforces the need to identify molecular and microenvironmental predictive markers, such as PRLR variants, D2 receptor expression, and miRNA profiles. Recognizing these new prognostic factors and their correlations with tumor clinical and radiological characteristics should help establish updated clinical practice guidelines for the management of prolactinoma and enable a more personalized therapeutic approach, thereby improving outcomes in patients with prolactinoma. Together, these findings may support a more personalized approach to dopamine agonist dose escalation and earlier consideration of multimodal therapy in high-risk patients.

## Figures and Tables

**Table 1:** Baseline characteristics of the study population (n = 85).

Variable ^1^	Value ^2^
Age, years, mean ± SD	41.52 ± 17.19
**Gender**	
Male, n (%)	54 (63.5)
Female, n (%)	31 (36.5)
Giant prolactinoma, n (%)	19 (22.4)
**Symptoms, n (%)**	
Headache	59/85 (69.4)
Visual field loss	30/85 (35.3)
Loss of libido	16/85 (18.8)
Erectile dysfunction	15/54 (27.8)
Irregular menes	21/31 (67.7)
Gallacthorrea	14/85 (16.5); gynecomastia 5/54 (9.3)
Central hypogonadism	65/85 (76.5)
Central hypothyroidism	21/85 (24.7)
Central hypocortisolism	19/85 (22.4)
**Baseline characteristics**	
Prolactin [ng/mL], Me (q1 − q3)	545.7 (173.2 − 2023.0)
Maximum diameter of the tumor [mm], Me (q1 − q3)	22.0 (13.0 − 36.0)
Tumor volume [mm^3^], Me (q1 − q3)	3364.65 (630.28 − 14,168.62)
**KNOSP classification**	
KNOSP grade 0, n (%)	15/85 (17.6)
KNOSP grade 1, n (%)	14/85 (16.5)
KNOSP grade 2, n (%)	20/85 (23.5)
KNOSP grade 3, n (%)	22/85 (25.9)
KNOSP grade 4, n (%)	14/85 (16.5)
Cavernous sinus invasion, n (%)	69/85 (81.2)
Suprasellar invasion, n (%)	30/85 (35.3)
Optic chiasm compression, n (%)	37/85 (44.6)
Sphenoid sinus invasion, n (%)	51/85 (61.4)
Cystic component, n (%)	19/85 (22.4)
Surgical treatment, n (%)	13/85 (15.5)
Total follow-up duration [months]; Me (q1 − q3)	52.0 (31.50 − 86.75)

**^1^** Continuous variables are present as median (Me) and interquartile range (IQR), quartile 1 − quartile 3 (q1 − q3), and categorical variables as frequency (n) and percentage (%). **^2^** The normality of the data distribution was evaluated using the Shapiro–Wilk test.

**Table 2:** Evaluation of the study population (n = 85) at 6 months, 12 months, and at the last follow-up visit.

Variable ^1^	Evaluation at 6 Months	Evaluation at 12 Months	Evaluation at Last Visit
	Value ^2^	Value ^2^	Value ^2^
Prolactin [ng/mL], Me (q1 − q3)	47.4 (14.45 − 241.35)	24.8 (6.85 − 61.15)	15.9 (8.45 − 55.25)
Prolactin normalization, n (%)	21/85 (24.7)	34/85 (40.0)	47/85 (55.3)
Δ Prolactin, Me (q1 − q3) ^3^	391.42 (45.05 − 1476.85)	457.97 (105.40 − 1995.95)	504.00 (117.05 − 1992.35)
Tumor shrinkage, n (%) ^4^	44/85 (51.8)	57/85 (67.1)	59/85 (69.4)
Tumor volume reduction (%), Me (q1 − q3)	23.15 (0.0 − 57.11)	43.70 (17.27 − 78.25)	63.48 (27.29 − 93.83)
≥50% reduction in tumor volume, n (%)	25/85 (29.4)	35/85 (41.2)	44/85 (51.8)
Maximum diameter of the tumor [mm], Me (q1 − q3)	18.00 (11.50 − 28.00)	18.00 (10.25 − 25.00)	15.00 (10.00 − 24.50)
Maximum tumor diameter reduction (%), Me (q1 − q3)	7.69 (0.00 − 26.36)	15.40 (0.00 − 33.33)	15.88 (0.00 − 43.15)
Maximum tumor diameter reduction ≥ 30%, n (%)	16/85 (18.8)	24/85 (28.2)	31/85 (36.5)
**Responders**	6/85 (7.1)	9/85 (10.6)	22/85 (25.9)
**Partial responders**	54/85 (63.5)	49/85 (57.6)	43/85 (50.6)
**Resistant to DA**	25/85 (29.4)	27/85 (31.8)	20/85 (23.5)
Maximum cabergoline dose [mg/week]; Me (q1 − q3) ^5^	1.0 (0.75 − 2.0)	1.0 (0.75 − 2.0)	1.0 (0.75 − 2.0)

**^1^** Continuous variables are present as median (Me) and interquartile range (IQR), quartile 1 − quartile 3 (q1 − q3), and categorical variables as frequency (n) and percentage (%). **^2^** The normality of the data distribution was evaluated using the Shapiro–Wilk test. **^3^** Delta (Δ) between baseline and follow-up PRL concentrations, calculated using the following formula: ΔPRL = (PRL follow-up − PRL baseline). **^4^** Any reduction in tumor volume or any increase in tumor volume. **^5^** The maximum cabergoline doses were defined as the highest dose used during the 6-month, 12-month, and last follow-up period and expressed as the median (Me) and the interquartile range (IQR).

**Table 3:** Comparison of the patients according to their response status at 6 months.

Variable ^1^	Non-Resistant (n = 60)	Resistant (n = 25)	*p*-Value ^2^
**Baseline Characteristics**			
Age [years]	44.36 + − 17.887	36.68 + − 13.074	0.119
**Male gender, n (%)**	**34/60 (56.7)**	**20/25 (80.0)**	**0.042**
**Giant prolactinoma, n (%)**	**8/60 (13.3)**	**11/25 (44.0)**	**0.002**
**Basal prolactin [ng/mL], Me (q1 − q3)**	**478.985 (175.00 − 1038.00)**	**2000.000 (470.00 − 4810.00)**	**0.012**
**Maximum diameter of tumor [mm], Me (q1 − q3)**	**19.0 (13.0 − 27.0)**	**30.0 (15.0 − 56.0)**	**0.012**
**Basal tumor volume [mm^3^], Me (q1 − q3)**	**2111.157 (600.831 − 8494.886)**	**10,306.540 (1429.430 − 49,553.504)**	**0.014**
**Symptoms, n (%)**			
Headache	39/60 (65.0)	20/25 (80.0)	0.171
Visual field loss	21/650 (35.0)	9/25 (36.0)	0.930
Loss of libido	10/60 (16.7)	6/25 (24.0)	0.544
**Invasion of the structures in MRI**			
Cavernous sinus invasion	49/60 (81.7)	20/25 (80.0)	1.000
**Suprasellar invasion**	**17/60 (28.3)**	**13/25 (52.0)**	**0.037**
Optic chiasm compression	23/60 (38.3)	14/25 (56.0)	0.134
Sphenoid sinus invasion	35/60 (58.3)	16/25 (64.0)	0.627
**Evaluation at 6 months**			
**Prolactin [ng/mL],** Me (q1 − q3)	**29.805 (8.20 − 116.60)**	**148.000 (86.00 − 479.00)**	**<0.001**
**Prolactin normalization, n (%)**	**20/60 (33.3)**	**1/25 (4.0)**	**0.004**
Δ Prolactin at 6 months [ng/mL], Me (q1 − q3) ^3^	235.185 (48.90 − 868.23)	1134.000 (155.60 − 4778.40)	0.054
**Maximum diameter of tumor [mm], Me (q1 − q3)**	**17.0 (11.0 − 24.5)**	**26.0 (15.0 − 41.0)**	**0.004**
Maximum tumor diameter reduction (%), Me (q1 − q3)	8.535 (0.00 − 28.57)	4.350 (0.00 − 20.00)	0.637
Maximum tumor diameter reduction ≥ 30%, n (%)	13/60 (21.7)	4/25 (16.0)	0.552
Tumor volume reduction (%)	20.145 (0.00 − 56.56)	29.040 (0.70 − 53.24)	0.502
**≥50% reduction in tumor volume, n (%)**	19/60 (31.7)	6/25 (24.0)	0.480
**Maximal cabergoline dose [mg/week] ^3^, Me (q1 − q3) ^4^**	**1.0 (0.5 − 1.0) (0.25 − 4)**	**2.0 (2.0 − 3.0) (2.0 − 4.0)**	**<0.001**
**Evaluation at 12 months**			
**Prolactin [ng/mL],** Me (q1 − q3)	**14.750 (5.30 − 30.00)**	**60.000 (25.00 − 267.00)**	**<0.001**
**Prolactin normalization, n (%)**	**30/60 (50.0)**	**4/25 (16.0)**	**0.004**
Δ Prolactin at 12 months [ng/mL], Me (q1 − q3) ^3^	435.500 (116.69 − 1036.00)	1048.000 (96.40 − 4798.70)	0.203
**Maximum diameter of tumor [mm], Me (q1 − q3)**	**14.75 (10.00 − 22.00)**	**22.00 (12.00 − 40.00)**	**0.018**
Maximum tumor diameter reduction (%), Me (q1 − q3)	14.835 (0.00 − 33.34)	16.667 (0.00 − 43.24)	0.888
Maximum tumor diameter reduction ≥ 30%, n (%)	17/60 (28.3)	7/25 (28.0)	0.975
Tumor volume reduction (%), Me (q1 − q3)	49.610 (18.75 − 77.13)	34.000 (14.29 − 80.49)	0.688
≥50% reduction in tumor volume, n (%)	26/60 (43.3)	9/25 (36.0)	0.531
**Maximal cabergoline dose [mg/week] ^4^, Me (q1 − q3) ^4^**	**1.0 (0.5 − 1.25) (0.25 − 4.0)**	**2.5 (2.0 − 3.5) (2.0 – 4.5)**	**<0.001**
**Last follow-up**			
**Prolactin [ng/mL], Me (q1 − q3)**	**13.150 (5.90 − 27.80)**	**32.100 (15.11 − 200.40)**	**0.005**
**Prolactin normalization, n (%)**	**38/60 (63.3)**	**9/25 (36.0)**	**0.021**
**Δ Prolactin at last visit [ng/mL], Me (q1 − q3) ^3^**	424.535 **(116.10 − 1023.58)**	**1950.000 (46.00 − 4684.89)**	**0.019**
**Maximum diameter of tumor [mm], Me (q1 − q3)**	**12.50 (9.0 − 22.0)**	**22.00 (15.0 − 38.0)**	**0.004**
Maximum tumor diameter reduction (%), Me (q1 − q3)	19.375 (0.00 − 42.86)	11.630 (0.00 − 43.24)	0.906
Tumor volume reduction (%), Me (q1 − q3)	61.535 (26.11 − 89.37)	72.780 (27.51 − 99.76)	0.385
**≥50% reduction in tumor volume, n (%)**	32/60 (53.3)	12/25 (48.0)	0.654
Total follow-up time [months], Me (q1 − q3)	50.50 (32.0 − 85.0)	61.00 (38.0 − 92.0)	0.386

**^1^** Continuous variables are presented as median (Me) and interquartile range (IQR), quartile 1 − quartile 3 (q1 − q3), and categorical variables as frequency (n) and percentage (%). **^2^** The normality of the data distribution was evaluated using the Shapiro–Wilk test. Categorical variables were compared using Fisher’s exact test, while continuous variables were analyzed with the Mann–Whitney U test. **^3^** Delta (Δ) between baseline and follow-up PRL concentrations, calculated using the following formula: ΔPRL = (PRL follow-up − PRL baseline). **^4^** The maximum cabergoline doses were defined as the highest dose used during the 6-month and 12-month follow-up period and expressed as the median (Me) and the interquartile range (IQR).

**Table 4:** Differences between males and females with prolactinomas.

Variable ^1^	Male (n = 54)	Female (n = 31)	*p*-Value ^2^
Age [years], mean ± SD	44.04 ± 16.9	37.9 ± 16.9	0.074
**Maximum tumor diameter at baseline [mm], Me (q1 − q3)**	**26.5 (18.0 − 47.0)**	**12.5 (9.75 − 19.5)**	**<0.001**
**Prolactin at baseline [ng/mL], Me (q1 − q3)**	**976.68 (364.9 − 4832.5)**	**204.35 (94.3 − 620.7)**	**<0.001**
Prolactin at 6 months [ng/mL], Me (q1 − q3)	66.6 (15.35 − 312.35)	44.6 (10.48 − 1256.6)	0.485
Prolactin at 12 months [ng/mL], Me (q1 − q3)	24.8 (8.23 − 65.48)	19.8 (5.2 − 60.23)	0.729
Prolactin at last visit [ng/mL], Me (q1; q3)	16.85 (8.48 − 65.3)	15.85 (7.8 − 55.13)	0.837
**Maximum tumor diameter at baseline [mm], Me (q1 − q3)**	**21 (12.75 − 26.25)**	**10.5 (5.93 − 15.8)**	**<0.001**
**Tumor volume at baseline [mm^3^], Me (q1 − q3)**	**7961.86 (2069.14 − 22,555.12)**	**605.67 (93.86 − 2227.39)**	**<0.001**
**Tumor volume at 12 months [mm^3^], Me (q1 − q3)**	**2333.16 (510.25 − 8906.44)**	**367.6 (72.77 − 1277.06)**	**<0.001**
**Resistant at 6 months**	**20/54 (37.0%)**	**5/31 (16.1%)**	**0.042**
**Resistant at 12 months**	**22/54 (40.7%)**	**5/31 (16.1%)**	**0.019**
**Resistant at last follow-up**	**16/54 (29.6%)**	**4/31 (12.9%)**	**0.080**
Total follow-up duration [months], Me (q1 − q3)	52.0 (30.0 − 80.0)	51.5 (33.5 − 138.75)	0.383
**Maximal cabergoline dose at 6 months [mg/week], Me (q1 − q3) ^3^**	**1.0 (1.0 − 2.0)**	**1.0 (0.5 − 1.5)**	**0.013**
**Maximal cabergoline dose at 12 months [mg/week], Me (q1 − q3) ^3^**	**1.5 (1.0 − 2.5)**	**1.0 (1.0 − 1.5)**	**0.002**

**^1^** Continuous variables are presented as median (Me) and interquartile range (IQR), quartile 1 − quartile 3 (q1 − q3), and categorical variables as frequency (n) and percentage (%). **^2^** The normality of the data distribution was evaluated using the Shapiro–Wilk test. Categorical variables were compared using Fisher’s exact test, while continuous variables were analyzed with the Mann–Whitney U test. **^3^** The maximum cabergoline doses were defined as the highest dose used during the 6-month and 12-month follow-up period and expressed as the median (Me) and the interquartile range (IQR).

**Table 5:** Univariate logistic regression predictors of resistance at 6 and 12 months.

Variable	OR	95% CI	*p*-Value
**6 months**			
**Sex (M = 1, W = 0)**	3.059	1.013–9.236	0.047
**Baseline maximal tumor diameter**	1.044	1.016–1.073	0.002
**12 months**			
**Sex (M = 1, W = 0)**	3.575	1.190–10.743	0.023
**Baseline maximal tumor diameter**	1.047	1.018–1.076	0.001

## Data Availability

Data supporting the findings of this study are available upon reasonable request from the corresponding author. Due to privacy and ethical restrictions, the datasets are not publicly accessible.

## Abbreviations

The following abbreviations are used in this manuscript:

DA	Dopamine Agonists
PRL	Prolactin concentration
ER	Estrogen Receptor
PRLR	Prolactin Receptor
PRL	Prolactin
MRI	Magnetic Resonance Imaging
GH	Growth Hormone
CT	Computed Tomography
aOR	Adjusted Odds Ratio
CI	Confidence Interval
MEN1	Multiple Endocrine Neoplasia type 1
AIP	Aryl hydrocarbon receptor Interacting Protein
NGFR	Nerve Growth Factor Receptor
